# Simultaneous upper limb melanoma and breast cancer related lymphedema management

**DOI:** 10.1080/23320885.2023.2182308

**Published:** 2023-02-24

**Authors:** Dimitrios Dionyssiou, Athanasios Papas, Avra Drougou, Athanasios Tsamaldoupis, Georgios Arsos, Efterpi Demiri

**Affiliations:** a Department of Plastic Surgery, School of Medicine, Aristotle University of Thessaloniki, Greece. GH Papageorgiou, Thessaloniki, Greece; b Dermatologist, Private Sector, Thessaloniki, Greece; c Department of Nuclear Medicine, School of Medicine, Aristotle University of Thessaloniki, Thessaloniki, Greece; d GH Papageorgiou, Thessaloniki, Greece

**Keywords:** Melanoma, breast cancer related lymphedema, sentinel lymph node biopsy, LVA

## Abstract

We present a rare case of a patient with malignant melanoma in the lymphedematous arm associated with breast cancer and its lymphedema management. Histology of previous lymphadenectomy and results of current lymphangiographies suggested the need for SLN biopsy, and simultaneously perform of distal LVAs to manage lymphedema.

## Introduction

Sentinel lymph node biopsy (SLNB) is the standard approach for the management of patients with cutaneous malignant melanoma (CMM) in whom there is an increased risk of regional node metastasis [1]. This method provides important prognostic information and permits the identification of patients with a positive sentinel lymph node who may be candidates for adjuvant therapy [1,2]. The efficient evaluation and feasibility of re-sentinel lymph node biopsies (re-SLNBs) has remained a challenge in the management of patients with previous SLNB or regional lymphatic clearance who are clinically lymph node negative.

We present a rare case of a female patient with a malignant melanoma in a lymphedematous upper limb, following mastectomy and axillary clearance, and its simultaneous melanoma and lymphedema treatment.

## Method

A 55-year-old Caucasian woman was referred to our department following a diagnosis of CMM after an excisional biopsy of an 8mm lesion in the middle lateral third of the left arm. The histology showed a Spitzoid melanoma with a Breslow’s thickness (BT) of 2.1mm, a mitotic rate of 3/mm^2^ and no ulceration or any other aggravating factors. In addition, she was complaining of the same left non-dominant extremity feeling of heaviness, three years after a total mastectomy and ipsilateral axillary lymph node dissection.

She had a history of left breast cancer, four years ago, for which she had undergone a tumorectomy. Due to a BC recurrence in a year, she underwent a neoadjuvant chemotherapy according to the standard of care related to that period, followed by left mastectomy with ipsilateral axillary lymph node dissection (ALND). The histopathologic examination showed a moderately differentiated invasive mucinous carcinoma of the breast (Elston and Ellis grade 2), with micropapillary features to a lesser extent. Three out of ten excised lymph nodes were positive, which led to a postoperative axillary region radiotherapy. BRCA gene mutation testing was positive and decided a contralateral prophylactic mastectomy along with laparoscopic prophylactic oophorectomy one year later.

On physical examination a scar from previous excisional biopsy was found in the lateral side middle third of the left arm. The patient mentioned that she had a feeling of heaviness characterized in subjective visual analogue scale 4 out of 5, and an oedematous discomfort of her left upper extremity (LUE) started after radiotherapy, but she denied pain or preceding erysipelas. As that was the first time she was examined for lymphedema, calculating arm volumes indirectly by circumference measurements and using the formula for a truncated cone [3] revealed a 4% or 122cc volume difference of the (LUE) (Table 1). She had a body mass index of 31,2 kgr/m^2^ and described herself as right-handed.

**Table 1.** Pre and post operative measurements of upper limb.

Pre-operative measurements of upper limbs circumference per 4cm										
Left	17	23	26	29.5	31	30	32	33	34	35
Right	18	22	25	28.5	30	30.5	32	34	34	35.5

Post-operative measurements of upper limbs circumference per 4cm										
Left	19	22	25	28	31	31	32	33	32	34
Right	19	22	25	28	31	32	34	34	36	37

**Figure 1.**
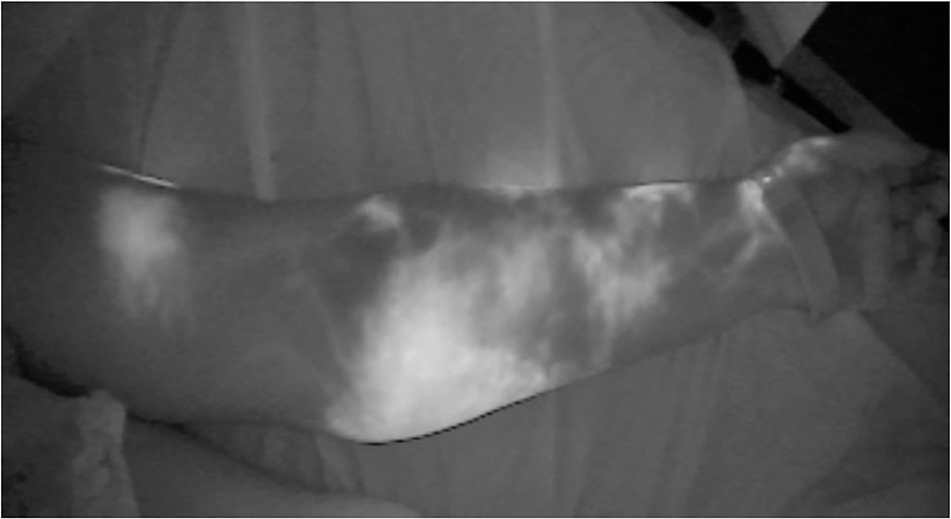
ICG lymphography showing dermal backflow, stardust pattern and diffuse lymphagiectasia, compatible with Yamamoto stage III/IV lymphedema.

Patient’s medical history consisted of thyroid nodules well controlled, dyslipidaemia, bilateral lower limb mild venous insufficiency, hypertension and rare episodes of sinus tachycardia, while the environmental impact at the disease concerned of social sun exposure mainly during summers, and no other skin cancers. Family history included breast cancer of her mother and prostate concer of her father. She was under letrozole, beta blocker and atorvastatin, had no known allergies and never smoked. Required PET/CT and brain MRI examinations for melanoma TNM staging demonstrated no pathology. Lymphatic system evaluation using indocyanine green (ICG) lymphangiography showed Yamamoto [4] stage IV arm dermal backflow (ADB), while hybrid SPECT/CT lymphoscintigraphy, revealed advanced dermal backflow in the left forearm, multiple in-transit lymph nodes in the left upper arm and remaining axillary lymph nodes with decreased function (Figure 1(a)). Findings of the right upper limb were normal.

Considering the small number of axillary lymph nodes that were excised from the left axilla during her previous lymphadenectomy and the SPECT/CT lymphoscintigraphy results, indicated remaining axillary lymph nodes and potential existence of sentinel lymph node biopsy. Under general anaesthesia a 2 cm wide local excision of the primary melanoma site, and a left axilla SLN biopsy were performed. SLNB was identified with a handheld gamma-camera probe radioisotopic lymphoscintigraphy, and Patent-V blue assisted dye injection at the site of primary melanoma.

**Figure 2.**
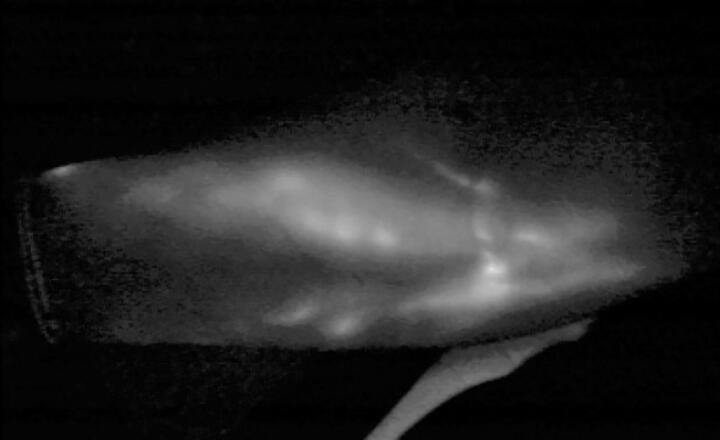
1 year post operative ICG showing linear lymphatic vessels and hypoplastic vessels with valve.

With the use of ICG lymphangiography, patent functional lymphatic channels were identified and marked distal of the dermal back flow area of the left forearm volar surface. The location for LVAs was chosen to be situated distally of the melanoma region, in order avoid any misconception of interference in a potential metastatic route at the central lymphatic circulation. Under microscopic view, three LVAs were performed in an end-to-end anastomosis fashion at the volar ulnar area of the forearm (Figure 2), and immediate restoration of the lymph outflow was confirmed using ICG fluoroscopy. Two weeks after the operation she was instructed to implement our LVA-lymphedema protocol daily manual lymphatic drainage and bandage for 10 days, following by a pressure garment Class II for twelve months during day time.

## Results

**Figure 3.**
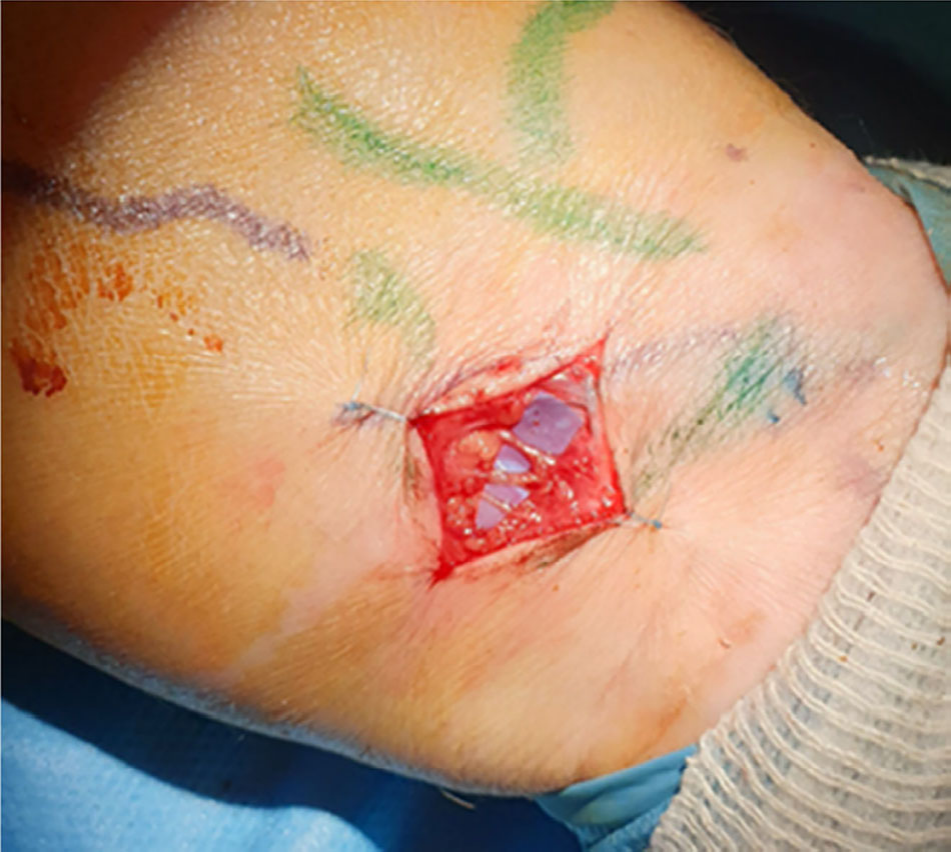
Lympho-venous anastomosis in the left forearm.

**Figure 4.**
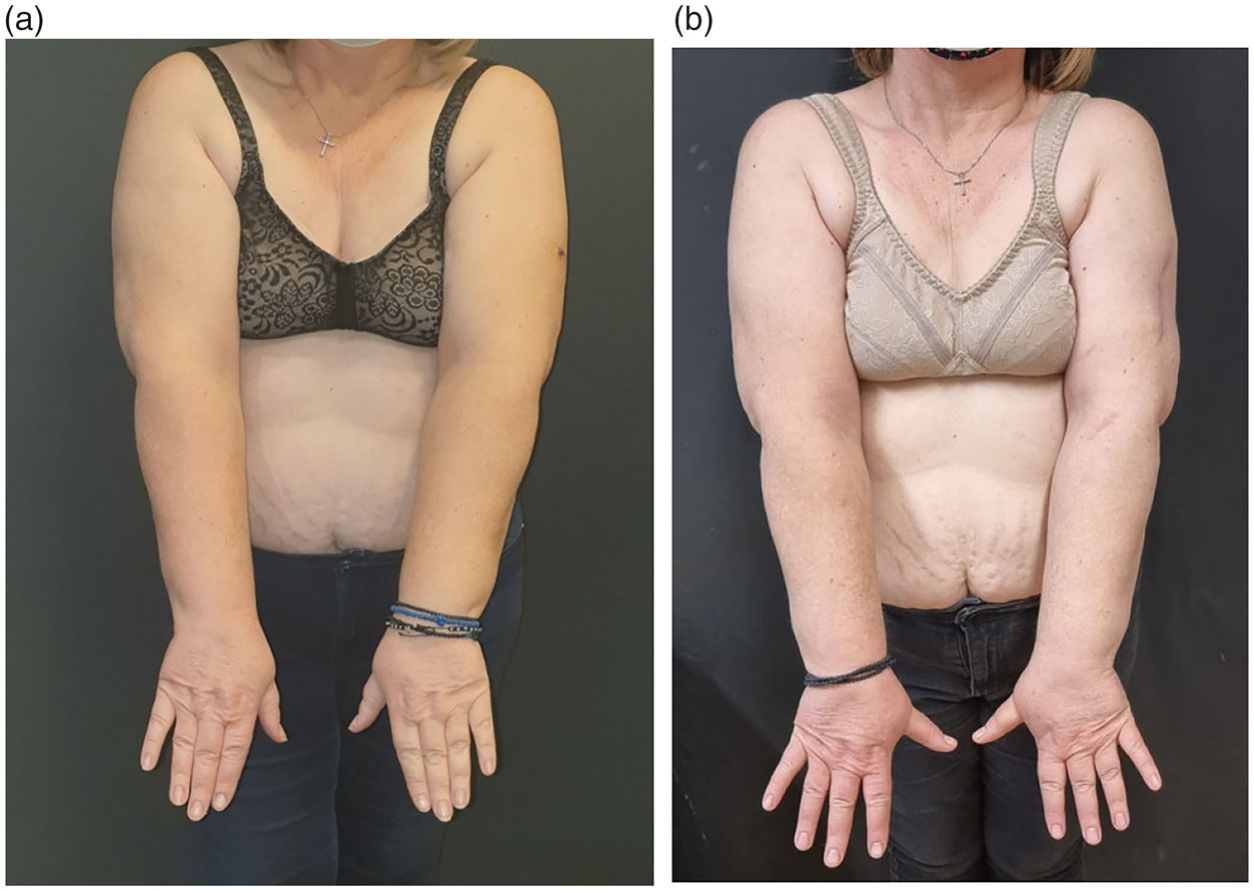
(a) Preoperative image of upper extremities. (b) Postoperative image of upper extremities.

The postoperative recovery was uneventful, the patient was discharged the third postoperative day, and followed the lymphedema protocol for 12 months. At the 12 months follow up she remains melanoma malignancy and breast cancer disease free. During her 12-month follow up, lymphedema was clinically improved, having softer the left upper arm without indentation or indication of lymph stasis, without feeling of heaviness, and a decreased upper limb volume from +4% to −9% (from +122cc to a -272cc). A new 12 months ICG lymphangiography revealed a downstaging of Yamamoto’s stage III/IV to stage II (Figure 3 and 4(b), Table 1). The patient was encouraged to gradually reduce the period of wearing the elastic garment during the day, and at the 18 months follow up the improved clinical situation remains unchanged.

## Discussion

Based on the recent American Joint Committee on Cancer (AJCC) staging manual of 2018 SLNB procedure should be considered for all patients with stage T1b melanoma and above, along with a subset of T1a patients with high-risk features such as such as mitotic rate ≥2/mm^2^, ulceration, lymphovascular invasion, or uncertain microstaging [1,2].

Since our patient had undergone a previous axillary clearance for breast cancer and the lymphatic flow may be altered due to fibrosis from surgery and adjuvant radiotherapy, it was uncertain the validity of a reoperative sentinel lymph node biopsy (SLNB) as a repeated axillary staging procedure. Considering that an axilla contains 20 to 30 lymph nodes [5], though the exact number varies between individuals, and that our patient had only 10 nodes excised during her previous dissection, we estimated that there would be remaining nodes in the axilla. Such finding was also confirmed by the SPECT/CT lymphoscintigraphy.

A review of literature [6,7] showed that repeat SLNB is feasible in patients after previous axillary staging. Sentinel lymph node identification was in 55% of the cases but aberrant lymphatic drainage was more frequent in this setting (43.2%) [7]. Others rase the success identification rate to 83% if <9 nodes were removed in the previous surgery [6]. In general localisation success appears to depend on the extent of previous axillary surgery. They also report that extra-axillary SLN biopsy localisation rates are higher than for primary SLN biopsy and emphasize the importance of preoperative lymphoscintigraphy to explore extra-axillary lymphatic drainage in this restaging setting. In our case only 10 lymph nodes were excised in the previous ALND and the remaining functional nodes were identified with lymphoscintigraphy. The sentinel node was identified in the axilla and was negative for metastasis.

When first the patient presented to our department she had an established left upper limb lymphedema but not diagnosed or managed before. If limb dominance is considered as a significant factor on the increased size, the dominant arm would expect to have a superior volume in comparison with the non-dominant contralateral extremity. According to Gebruers, the relatively small 4% volume difference between the two arms could be defined as 7.3%+/-3.3%, while the the −9% reduction (from + 122cc to -272cc) after LVA and adjuvant lymphedema treatment at 12 months can also be explained similarly as the operated arm was the non dominant one (Gebruers et al. 2007) [8].

Goggins et al. observed an elevated risk (46%) of a second CMM among young breast cancer patients. Also women who underwent radiation therapy exhibited a 42% increased risk for CMM. The risks of BC among female CMM survivors and CMM among BC survivors were also elevated, albeit to a much lesser degree (overall, 11% and 16%, respectively) [9].

Another study [10] found that female BC survivors younger than 45 years old had a 1.38 relative risk (or 38% increase from the general population) of developing CMM as a second cancer. Female breast cancer patients 45 years and older had a 12% increase in the risk for being diagnosed with melanoma [11].

Ho et al. [12] also demonstrated that there is a bidirectional correlation between malignant melanoma and breast carcinoma. They recommended increased awareness among clinicians leading to more detailed surveillance of both second primary tumours, as well as all CMM patients with a family history of BC should be referred to a breast clinic. Women above the age of 40 with CMM should undergo annual mammography and those less than 40 may be better evaluated with a breast MRI. All breast cancer patients should be made aware of the significance of changing moles and those with suspicious lesions referred to a dermatologist for evaluation.

## Conclusion

ReSLNB is feasible after previous ALND and is recommended in patients with cancer where this procedure is indicated. Because the altered lymphatic drainage can be detected by lymphoscintigraphy, the radioisotopic lymphoscintigraphy, should be used. Information gained from the previously excised lymph nodes and lymphangiography provide an inside view regarding the presence of residual functional lymph nodes and the possibility of performing a SLN biopsy. In addition, the simultaneous restoration of lymphedema is also feasible with super-microsurgical LVA technique.
